# Can Information Change Public Support for Aid?

**DOI:** 10.1080/00220388.2018.1493194

**Published:** 2018-07-16

**Authors:** Terence Wood

**Affiliations:** The Development Policy Centre, The Crawford School, The Australian National University, Canberra, Australia

## Abstract

Donor country publics typically know little about how much aid their governments give. This paper reports on three experiments conducted in Australia designed to study whether providing accurate information on government giving changes people’s views about aid. Treating participants by showing them how little Australia gives or by showing declining generosity has little effect. However, contrasting Australian aid cuts with increases in the United Kingdom raises support for aid substantially. Motivated reasoning likely explains the broad absence of findings in the first two treatments. Concern with international norms and perceptions likely explains the efficacy of the third treatment.

## 1. Introduction

A growing body of academic work exists on the socio-demographic and political traits associated with public support for government aid in donor countries (for recent examples see, Cheng & Smyth, 2016; Heinrich, Kobayashi, & Bryant, 2016; Paxton & Knack, 2012). One motivation for this work is the fact that greater levels of public support for aid are associated with donor countries devoting a larger share of their Gross National Income (GNI) to aid (Stern, 1998, p. 3; Milner & Tingley, 2010, p. 216, 2011, p. 55). This work has identified a suite of traits (including left-wing political views, age, and education) that are typically associated with support for aid (Wood, 2015, pp. 3–6). Yet, while this work has helped to build a good picture of people’s existing views of foreign aid, to-date there has been much less academic work looking at whether existing views about government aid can be changed. The aid-related work that does exist typically focuses on donations to NGOs (for example, Clark, Garces-Ozanne, & Knowles, 2018), or covers aid only in passing (for example, Gilens, 2001), or focuses only on the provision of information on matters other than aid (for example, Nair, in press). Such work provides a helpful starting point in learning how information can change support for aid. However, there remains much scope to build on it.

In the paper that follows I build on existing work by reporting on research that focuses specifically on government aid, and which focuses on the effect of providing the public with accurate information on how generous their government is in its aid efforts. (For the purpose of this paper ‘generosity’ is quantified as government aid relative to GNI or to government spending.) This is the first ever systematic, academic research to focus on the effect of information on government donor generosity on support for aid.

The provision of information on generosity is a logical place to start in testing whether information more broadly can change public support for government aid. In most OECD donor countries members of the public overestimate how generous their government is (German, Helmich, Randell, & Smillie, 1998; Hudson & vanHeerde-Hudson, 2012). Yet, correct information is easily obtained from OECD databases and similar sources (see OECD DAC, 2018). And there are good practical and theoretical reasons to be interested in whether public opinion about aid can be shifted by this information. Practically, given the link between public support for aid and donor generosity, aid advocates have an interest in knowing what can change opinion, as this may provide a pathway to aid increases. Campaigns in support of aid are often vigorous, and while some campaigners may conduct their own message testing, there is little evidence to suggest this is done in a rigorous way, or that campaigning is guided by a systematic understanding of what is likely to work. And at present there is an insufficient body of academic work to allow campaigners to draw from the research of social scientists. More theoretically, studying the impact of information on people’s support for government aid can help identify whether the factors that shape people’s views about public policy involving international development are similar to the factors that shape their private actions in the form of donations to aid NGOs (for example, Clark et al., 2018). Furthermore, increasingly sophisticated work is being undertaken to study the impact of information on peoples’ views about other aspects of international relations (Herrmann, 2017; Kertzer & Zeitzoff, 2017; Nyhan & Reifler, 2010). Better knowledge of the impact of information on the formation of views about aid has the potential to contribute to this broader area of study.

The research detailed in this paper involves three separate experiments conducted in Australia. Australia serves as a good country case for this research for four reasons. First, Australians are typical of donor publics in OECD countries in that most Australians overestimate how much aid their government gives (Burkot & Wood, 2015, p. 9). Second, Australia is a typical donor country in terms of aid generosity. The Australian government is neither unusually generous, nor unusually reluctant to give aid. In 2015 its rank amongst OECD aid donors based on aid as a share of GNI was one position higher than the median donor; in 2016 (the most recent year data are available for) it was one position lower than the median donor (Development Policy Centre, 2018). Third, by the standards of donor countries, the Australian public appear neither unusually supportive of aid, nor unusually opposed to it (Paxton & Knack, 2012, p. 179). Fourth, as I detail further below, recent changes in levels of Australian aid provide particularly useful comparators for the second and third experiments. Although it is not possible to guarantee the external validity of findings beyond Australia, because there is nothing obviously unusual about Australia as a donor country, there is no strong prima facie cause for believing that learnings from Australia will not be applicable elsewhere. Australia, it should also be noted, is a non-trivial donor in an absolute sense. In terms of total US dollars given, the Australian government was in the top half of OECD donor countries every year from 2010 to 2016 and was in the top third of donors for three of these years (OECD DAC, 2018).

To test the effect of the provision of information on aid generosity I conducted three separate survey experiments over three consecutive weeks. Each experiment had different participants. As survey experiments, each of the experiments involved the random allocation of participants into treatment and control groups. Treatment and control groups were provided with different information about aid but were asked the same survey questions pertaining to their views about aid volume. Each experiment was conducted online and involved a broadly nationally representative sample of approximately 1000 voting-aged Australians. All of the experiments had the same control: a simple question about whether the Australian government spends too much or too little on foreign aid. In the first experiment, the treatment group received this question but with additional information on Australian aid as a share of Australian federal government spending. This was information that, in effect, showed that the Australian government devotes only a very small share of federal spending to aid. In the second experiment, the treatment involved showing people a chart of decreasing Australian government aid generosity over time (as measured by aid as a share of GNI). In the third experiment, respondents in the treatment group were provided information on recent Australian aid cuts as well as information on recent increases in aid in the United Kingdom. The first experiment was designed to test the impact of the provision of accurate information on generosity to a public that often overestimates how much aid their country gives. The second experiment was designed to test the impact of providing a frame of reference which placed current spending in perspective by detailing past efforts. The third experiment was designed to provide a frame of reference involving a tangible example of what another, relevant, comparator country was doing.

My key findings are that the first treatment had no effect whatsoever and that the second treatment’s effect was very modest. However, I found that the third treatment had effects that were not only statistically significant but also significant in a substantive sense. Taken together, these results show that information on donor generosity can shift the public’s views about aid; however, the processes involved are not as simple as people updating their views in the wake of information that corrects previously held mistaken beliefs.

The rest of this paper is structured as follows. First, in three separate subsections, I describe each of the three treatments and draw on existing work from the study of public opinion, as well as other relevant information, to explain why the treatment was included. I also examine why the treatment in question might be expected to work, and why it might not. I then provide a description of relevant methodological information before detailing my findings. After my findings, I conclude by discussing the findings, their explanations and ramifications.

## 2. The three experiments, and their theoretical and empirical basis

As I discuss in more detail in the methods section below, subjects were randomly assigned to treatment and control groups in all three experiments. The control group received the following question in all three experiments:

Every year the Australian government provides aid money to poorer countries. Which of the following options best reflects your opinion about aid spending:

(a) the Australian government gives too much aid;

(b) the Australian government gives about the right amount of aid;

(c) the Australian government does not give enough aid;

(d) I don’t know.

Notably, in the question asked of the control group, no information was provided on how much aid Australia currently gives. Nor was any reference point provided to put current giving in perspective. Both in the provision of very limited information, and in its wording more generally, this question is similar to those used in much of the existing, non-experimental work on public opinion about aid (see, for example, Chong & Gradstein, 2008; Diven & Constantelos, 2009; Paxton & Knack, 2012; Wood, Humphrey Cifuentes, & Pryke, 2016).

### 2.1. Providing accurate information on aid effort

In Experiment 1 the treatment group was asked a question that was identically worded to the control question except for one additional item of information. This was information to do with the share of federal spending devoted to foreign aid in Australia. The modified question was:

Every year the Australian government provides aid money to poorer countries. Currently just under 1 dollar out of every 100 dollars of federal spending is given as aid. Which of the following options best reflects your opinion about aid spending: [identical response categories to those used in the control question were provided].^1^

Existing survey data from Australia provided an obvious reason for anticipating that this treatment would be effective in increasing in the proportion of respondents who thought Australia did not give enough aid and decreasing the proportion who thought Australia gave too much. Existing data show that, when asked, many Australians overestimate how much aid Australia gives by a significant margin. For example, when the survey company Essential Media asked a representative sample of Australians what they thought the share of the federal budget spent on aid was in its 11 July 2011 omnibus survey, of those who thought they knew, 75 per cent overestimated the amount and 46 per cent overestimated it substantially (Burkot & Wood, 2015, p. 9). In 2015, when Essential Media repeated the exercise in the midst of heated public debate about the largest aid cuts in Australia’s history, accurate estimates increased somewhat; however, 32 per cent of those survey respondents who thought they knew the size of the aid budget still overestimated it by at least a magnitude of five (Burkot & Wood, 2015, p. 10). In the same surveys, Essential Media also asked survey respondents for their views about whether Australia gave too much aid or not enough aid. There was a clear relationship between overestimation of the aid budget and the belief that Australia gave too much aid. In the 2015 survey, 66 per cent of those respondents who thought Australia devoted more than 5 per cent of federal spending to aid thought it gave too much. Only 26 per cent of those who thought it gave 1 per cent or less (the correct response) thought it gave too much aid (Burkot & Wood, 2015, p. 11). Many Australians overestimate how much aid Australia gives, and overestimation of aid volumes is associated with a desire to reduce aid. Given these facts, a simple model of cognition in which humans update their beliefs on the basis of corrective information would suggest that treatment, in the form of accurate information on aid budgets, ought to change opinions.

Some existing empirical work on support for aid provides additional grounds for anticipating that the treatment used in Experiment 1 would be effective. In a study conducted by the Kaiser Family Foundation, United States-based participants were asked in telephone interviews what they thought aid was as a share of United States federal spending. They were then asked if they thought the United States gave too much or too little aid. As in Australia, participants in the United States study typically overestimated aid effort. After the initial round of questions, participants were then told how much aid the United States actually gave and were asked again whether they thought the United States gave too much or too little aid. In between being asked the first time and being asked the final time, the share of respondents who thought the United States gave too much aid dropped from 56 per cent to 28 per cent (Julio, Firth, & Brodie, 2015). In other recent work, Nair (in press) found that survey participants in the United States who were shown how wealthy they were compared to most of the world’s population, were, on average, considerably more supportive of aid than their counterparts in a control group, who were not provided this information. In this instance, the information Nair provided did not actually pertain to donors, or to donor generosity, but the study still showed that some forms of information, at least, can shift views about aid spending. Similarly, experimental work on donations to NGOs indicates various forms of information have an impact on people’s willingness to give to NGOs undertaking aid work. Brañas-Garza (2006) finds that informing experiment subjects about recipient poverty increases donations. Hansen, Kergozou, Knowles, and Thorsnes (2014) find that information on recipient country characteristics plays a role in influencing which countries experimental subjects choose to donate to when choosing between countries, as do Etang, Fielding, and Knowles (2012). Similarly, Karlan and Wood (2014) find that information about project efficacy increases the propensity of donors who had donated large amounts previously to give again.

However, there are also reasons to believe that treating people by providing them with correct information about the size of the Australian government aid budget might not have the anticipated impact. In the Australian surveys cited earlier, people who overestimated the aid budget were more likely to want it reduced; however, it was also the case that significant proportions of people who said they did not know the size of the aid budget also wanted it reduced (43% of those who answered ‘don’t know’ in 2015 wanted the budget cut) (Burkot & Wood, 2015, p. 11). Some people are clearly able to form opinions about aid even when fully aware of how little they know. If this is representative of a broader absence of interest in evidence when forming beliefs, it may be the case that providing corrective information will not change people’s views. Moreover, the Kaiser Foundation study cited above went further than simply providing people with information about aid spending: it also actively corrected people whose estimates were wrong. Possibly the effect captured by this approach is not that of information on donor generosity per se but is instead the effect of feelings associated with being proven wrong by the interviewer. If this is the case the findings may not be generalisable to situations where the only treatment is information, rather than information and embarrassment or similar feelings associated with being shown to be incorrect by an interviewer. (Because my interest was solely in the impact of correct information on donor generosity, rather than the effects of being proven wrong by an interviewer, I did not seek to emulate the Kaiser approach).^2^

Also, while – as detailed above – some existing aid-related experimental work involving the study of donations to NGOs provides cause to believe information can shift people’s generosity with respect to others in need, findings in this area are ambiguous. Although Etang, Fielding and Knowles (2012) find that information has some effect on which countries people chose to donate to when afforded the ability to allocate money between countries, they do not find any evidence that country traits influence subjects’ propensity to donate overall. (This is also the central finding of Etang, Fielding, and Knowles [2010].) And while Karlan and Wood (2014) found that information on project efficacy affected the propensity to donate of a small subset of participants, they found no treatment effect for the group as a whole. Similarly, Clark, Garces-Ozanne and Knowles (2018) did not find clear evidence that framing appeals for donations in terms of solutions was more efficacious in soliciting donations than framing appeals for donations in terms of problems.

There is also evidence from other, non-aid-related academic work that suggests that providing people with additional information about contentious issues does not change attitudes, or that the effects of such information provision are inconsistent at best. For example, in reviewing a range of experiments in which he provided information on immigration-related matters to United States-based participants, Sides (2007, p. 14) concluded that, ‘on average, then, providing correct information does not change attitudes toward immigration’.^3^ Likewise, survey experiments run by YouGov showed that providing people in the United States with detailed information on what their taxes were spent on had little impact on their views about tax levels or government spending levels (YouGov US, 2011). In another instance, Nyhan and Reifler (2010, p. 315) found inconsistent treatment effects in their newspaper experiments on views about the Iraq War, with information’s effects tending to be higher when it fit with respondents’ prior beliefs.

### 2.2. Providing information on aid trends over time

Another reason why treating people by simply providing information about current aid spending on its own might not have an effect is that this information on its own may not provide people with a sufficient frame of reference for evaluating whether present aid spending is adequate, inadequate, or more than adequate. Plausibly, for information to shift views it might need to be set in a context that shows how much spending is reasonable or feasible. To address this possibility, in the second experiment I provided participants in the treatment group both with information on current aid levels and with a point of reference: how much aid Australia has provided in recent decades (as a share of GNI). Specifically, I asked the following question, accompanied by a chart showing change over time. (The chart can be viewed in the Supplementary Material for this article).^4^

Every year the Australian government provides aid money to poorer countries. Over time, compared to the size of Australia’s economy, Australia’s aid budget has become smaller.

This chart compares Australian aid to Australia’s Gross National Income (a standard measure of the size of an economy). In 1971 Australia gave 45 cents of aid for every 100 dollars of Gross National Income. In 2015 it gave 25 cents. Which of the following options best reflects your opinion about aid spending: [identical response categories to those used in the control question were provided].

Existing research provides some cause for believing that information on past spending could be a useful point of reference, guiding people about what is possible, or normal, when it comes to aid spending. The influence of reference points on preference formation is well established in psychology (see, for example, discussion in Kahneman, 1992). Moreover, one existing published, experimental, academic study that includes a test of the efficacy of a simple information treatment in shifting views about aid (that of Gilens, 2001) drew on a treatment which provided information on declining trends in aid as a share of federal spending in the United States. (This was done as part of a study on a range of spending areas, not just aid.) The question Gilens used was:

The second story is about a new report that was just released about American foreign aid to help other countries. It said that the amount of money we spend for foreign aid has been going down and now makes up less than one cent of every dollar that the federal government spends. Have you heard about this story? (Gilens, 2001, p. 381) (Gilens then went on to ask participants’ views on aid spending.)

Gilens’s key experimental finding to do with aid was that the treatment he used had an impact (in the expected direction) on views about desired aid spending, although the effect was most clearly discernible amongst people who were more generally knowledgeable about political issues (Gilens, 2001, p. 386).

### 2.3. Comparison with the United Kingdom

The final treatment involved a different point of comparison: the United Kingdom. The treatment question was:

Every year the Australian government provides aid money to poorer countries. Since 2013 Australia has reduced the amount of aid it gives. At the same time, some countries, such as the United Kingdom, have increased the aid they give. The United Kingdom now gives about 70 cents out of every $100 of its Gross National Income as aid (Gross National Income is a standard measure of the size of a country’s economy). By comparison Australia gives 25 cents out if every $100 of its Gross National Income as aid. Considering this, which of the following options best reflects your opinion about aid spending: [identical response categories to those used in the control question were provided].

Recent non-aid-related research provides reasons to believe that comparisons with another member of the global community might have an effect on views about aid. Work in psychology, behavioural economics, and political science has shown that human beings’ propensity to confirm with norms can shape behaviour and views (Behavioural Insights Team, 2012; Gerber, Green, & Larimer, 2008; Tankard & Paluck, 2016). Moreover, recent work in political science has demonstrated the importance of peer effects in shaping people’s views about foreign policy issues (Kertzer & Zeitzoff, 2017). In a closely related area, experimental study of people’s donations to NGOs has found that people are more likely to donate when others are made aware of their giving (Andreoni & Petrie, 2004; Ariely, Bracha, & Meier, 2009). Similarly, players in dictator game experiments have been found to be more generous to their counterparts when their choices are known to others (Dana, Cain, & Dawes, 2006).

Taken together, the above research suggests that knowledge of how generous their government is in contrast to a relevant comparator could change people’s views of how generous they think their government should be on the international stage, either because they fear something akin to social sanctions for being seen to be a poor performer or because they see some form of positive benefit associated with their country being seen as a generous giver.

Because of this, it is plausible that a comparison with the more generous United Kingdom might alter Australians’ views about aid. The United Kingdom was chosen as a comparator because trends in aid in the United Kingdom contrast clearly with those in Australia. It was also chosen because it is a country to which Australia still has clear ties. English is the official language of both countries, Australia is a former colony, Australia is a member of the Commonwealth and for many Australians the United Kingdom continues to provide some form of cultural reference point (Meaney, 2001). The United Kingdom’s recent aid increases are not representative of the behaviour of the global aid community as a whole, but survey participants were unlikely to know this. They were, on the other hand, likely to view the United Kingdom as relevant.

Even so, observing an effect with this treatment was far from guaranteed. Most of the empirical work on norm-conforming behaviour has been based around the behaviour of individuals who modify their own personal behaviour to conform with a group of reference (typically their peers or community or compatriots). Unsurprisingly, norms have been shown to be at their most powerful in shaping behaviour when people identify with the relevant normative community being invoked (Tankard & Paluck, 2016, p. 197). Experimental research has found that norm-related behaviour modification is sensitive to the type of communities used as frames of reference (Behavioural Insights Team, 2012, p. 23). For the United Kingdom treatment to work on Australians’ views about aid, causal processes would be required that involved individuals’ being concerned about how their country was performing vis-à-vis a perceived international community. In this instance, a necessary condition for the treatment to work (at least if it were to work by normative mechanisms) would be for Australians to feel that the United Kingdom was part of a shared normative community with Australia. Moreover, Australians would need to care about Australia conforming to behaviour within this community in a manner analogous to the way that individuals care about their own personal conformity with more localised communities. It is true that some of the experimental work on propensity to donate to NGOs when others were made aware of donations involved experiments in which participants never met other players. This was also the case in the experimental play in the dictator game described above. The fact that behaviours were still modified in spite of this suggests that – in instances – people’s desire to be seen as good can be found even outside more clearly defined normative communities. Nevertheless, even taking this into account, the differences between people and governments, and between real communities and an imagined international community, are such that the efficacy of treating Australians by comparing their country to the United Kingdom could not be taken as a given in advance.

## 3. Methods and data

The three experiments were undertaken in three different weeks by different participants.^5^ In each week, survey participants were randomly assigned to receive the control question or that week’s treatment question. All of the survey experiments were conducted by the commercial survey firm Essential Media as part of weekly omnibus surveys. All experiments were completed online and had total sample sizes of about 1000 people. The surveys were conducted between November and December 2015. No other survey experiments were conducted by Essential Media in the weeks the aid survey experiments were run. There were no major media events involving Australian aid over the period in which the surveys were held. Although the survey process used by Essential Media was conducted online, it did not involve self-selection. Rather, the samples were randomly drawn from a large population (over 100,000 people) of participants in Essential Media’s survey pool. The pool of potentially sampled participants was large and diverse, and a reasonable approximation of the Australian population. I was also provided with data from responses to the socio-demographic and political questions asked in the omnibus surveys, enabling me to test whether the treatment and control groups were balanced in these aspects. A breakdown of these socio-demographic and political traits, and their balance across the control and treatment groups for all three experiments are provided in Table 1 in the Supplementary Materials.

**Table 1.** Results from aid as share of federal spending treatment

	Proportion control	Proportion treatment	Difference	Std. Error of diff	P-value (2 tailed)
Too much	0.39	0.40	−0.01	0.03	0.81
About Right	0.32	0.30	0.01	0.03	0.62
Not Enough	0.13	0.15	−0.03	0.02	0.25
Don’t know	0.16	0.14	0.02	0.02	0.41

As can be seen in the table in the Supplementary Materials, randomisation produced reasonably comparable groups, particularly in the second and third experiments. However, to control for any variations that could occur, in the results section, in addition to simple tests between treatment and control groups, I also present analysis using logistic regressions controlling for variation between the treatment and control groups. I was provided with survey weights designed to make the samples demographically representative of the Australian population. I did not apply these weights to the simple tests comparing treatments and control groups. However, I have applied survey weights to all regression analysis.

## 4. Results

In the following section I work through each of the three experiments, first presenting basic findings, before reporting on logistic regressions in which the socio-demographic variables identified above were controlled for, and in which survey weights were applied.

### 4.1. Providing accurate information on aid generosity

Table 1 details the proportion of respondents in the treatment and control groups who gave each of the possible replies in Experiment 1 (in which the treatment group was told how small Australian aid was as a share of Federal spending). While the treatment reduced the proportion of respondents who answered ‘don’t know’ slightly and increased the proportion of respondents who answered ‘not enough’ to the question about Australian aid levels, the changes were not statistically significant. Even if one is willing to ignore statistical insignificance, they are very modest in a substantive sense.

Table 2 shows the results of two logistic regressions run with control variables added. The controls were chosen on the basis that these variables have been found to be associated with attitudes to aid, either in other Australian work or in international work. In the first logistic regression, the dependent variable was binary and coded one if the respondent answered ‘not enough’. In the second model the dependent variable was binary and coded one if the respondent answered ‘too much’.^6^ Although some of the control variables were statistically significant predictors of attitudes to aid volumes, the treatment variable was not.^7^

**Table 2.** Logistic regressions with aid as share of federal spending treatment and controls

	Not enough aid	Too much aid
Treated	1.16	0.98
	(0.53)	(0.88)
Male	0.66*	0.95
	(0.08)	(0.74)
Over 35	1.95**	2.04***
	(0.02)	(0.00)
Urban	1.02	0.89
	(0.93)	(0.50)
Income (ln)	0.99	0.90
	(0.95)	(0.39)
Academic Education	3.20***	0.52***
	(0.00)	(0.00)
Party		
Labor	3.31***	0.91
	(0.00)	(0.60)
Greens	17.74***	0.29***
	(0.00)	(0.00)
Other	2.20*	1.53
	(0.08)	(0.12)
Don’t Know	2.34*	0.56**
	(0.06)	(0.03)
Constant	0.02***	0.99
	(0.00)	(0.98)
n	815	815

*Notes*: Odds ratios and p-values shown; regressions run with survey weights and robust SEs. *p < 0.1, **p < 0.05, ***p < 0.01; in all regressions the omitted party is the (centre-right) Coalition; academic education is a binary variable, coded one if the respondent has completed a degree from an academic tertiary institution (vocational tertiary education along with having no tertiary education is coded as zero).

### 4.2. Providing information on aid trends over time

Table 3 details the proportion of respondents in the treatment and control groups who gave each of the possible replies in Experiment 2 (in which the treatment group was told about trends in Australian aid spending). The comparisons show that the treatment effect is very small for three of the response categories: ‘about right’, ‘not enough’, and ‘don’t know’. However, the effect on people who believe too much aid is given is somewhat larger. As can be seen in this basic comparison, even the treatment’s effect on the proportion of people who think Australia gives too much aid is not statistically significant. However, because there is a clear expected direction of effect (a reduction), running the tests with a one-tailed test might be more appropriate. If this is done, the p-value is 0.09, suggesting this is a finding that we can be somewhat confident in if we wish to be lenient with regards to conventions of statistical significance.

**Table 3.** Results from aid trends over time treatment

Response	Proportion control	Proportion treatment	Difference	Std. Error of diff	P-value (2 tailed)
Too much	0.40	0.36	0.04	0.03	0.18
About Right	0.31	0.33	−0.02	0.03	0.57
Not Enough	0.16	0.18	−0.02	0.02	0.38
Don’t know	0.13	0.13	0.00	0.02	0.86

Table 4 reports the results of logistic regressions in which the same controls are used as in Experiment 1 and in which binary dependent variables were coded as in Experiment 1.

**Table 4.** Logistic regressions with aid trends treatment and controls

	Not enough aid	Too much aid
Treated	1.27	0.73**
	(0.26)	(0.05)
Male	1.15	1.27
	(0.53)	(0.13)
Over 35	0.83	2.09***
	(0.44)	(0.00)
Urban	1.60*	0.55***
	(0.07)	(0.00)
Income (ln)	0.86	0.91
	(0.29)	(0.46)
Academic education	2.67***	0.54***
	(0.00)	(0.00)
Party		
Labor	3.42***	0.67**
	(0.00)	(0.04)
Greens	8.34***	0.31***
	(0.00)	(0.00)
Don’t Know	1.12	0.59*
	(0.83)	(0.10)
Other	3.97***	1.05
	(0.00)	(0.85)
Constant	0.06***	1.31
	(0.00)	(0.64)
n	842	842

*Notes*: Odds ratios and p-values shown; regressions run with survey weights and robust SEs. *p < 0.1, **p < 0.05, ***p < 0.01.

With the control variables added, the impact of the treatment on the proportion of respondents who think Australia gives too much aid now becomes statistically significant at the 5 per cent level. The change is a product of both including survey weighting (which reduces the p-value slightly), and control variables improving the precision of the estimate, particularly once political parties are controlled for.^8^

**Figure 1.**
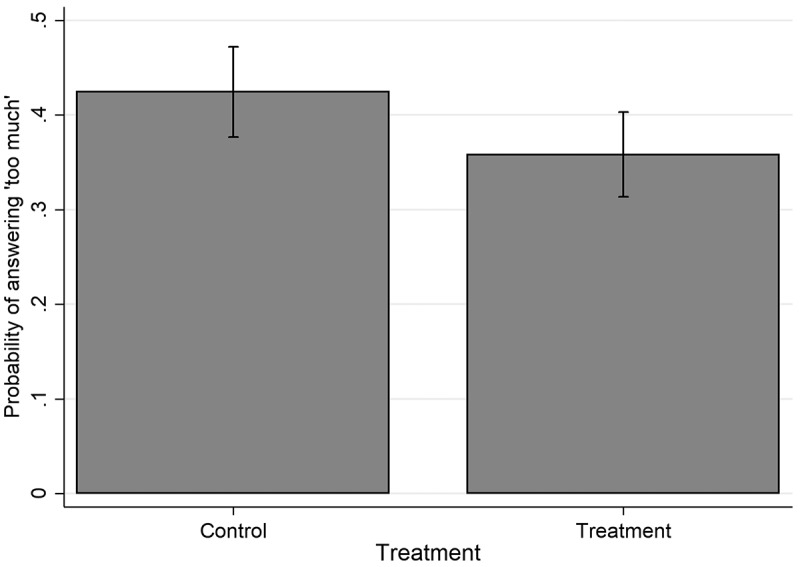
Estimated marginal effect of treatment with information on aid trends.

Figure 1 plots the predicted marginal effect of the treatment estimated from the results of the logistic regression.^9^ It is provided to give a sense of the substantive effect of Experiment 2’s treatment on the proportion of Australians who think Australia gives too much aid once controls have been added. The effect has become statistically significant, but it is confined to one answer category and its substantive effect is still modest.

### 4.3. Comparing Australia with the United Kingdom

In the final experiment, the treatment involved a comparison between increases in aid in the United Kingdom and recent aid cuts in Australia. Table 5 shows the proportion of respondents in the treatment and control groups who gave each of the possible replies to Experiment 3. The contrast with the previous experiments is striking. The treatment has clearly had an impact: the percentage of respondents who think Australia gives too much aid is over 10 percentage points lower in the treatment group than it is in the control group. The percentage of respondents who think Australia does not give enough aid is almost 10 percentage points higher. For the categories ‘too much’ and ‘not enough’ the differences are both statistically significant and substantively significant.

**Table 5.** Results from United Kingdom comparison treatment

Response	Proportion control	Proportion treatment	Difference	Std. Error of diff	P-value (2 tailed)
Too much	0.40	0.28	0.12	0.03	0.00
About Right	0.31	0.33	−0.02	0.03	0.56
Not Enough	0.14	0.22	−0.08	0.02	0.00
Don’t know	0.14	0.16	−0.02	0.02	0.44

Table 6 reports the results of logistic regressions similar to those used in the previous two experiments. Table 6 shows that the key findings from the simple tests of proportions hold even after the inclusion of control variables.

**Table 6.** Logistic regressions with United Kingdom treatment and controls

	Not enough aid	Too much aid
Treatment	1.62**	0.54***
	(0.03)	(0.00)
Male	1.04	1.40**
	(0.86)	(0.05)
Over 35	1.13	1.32
	(0.62)	(0.17)
Urban	1.77**	0.77
	(0.03)	(0.15)
Income (ln)	0.89	0.82
	(0.50)	(0.15)
Academic education	2.10***	0.45***
	(0.00)	(0.00)
Labor	1.88**	0.74
	(0.02)	(0.13)
Greens	8.84***	0.15***
	(0.00)	(0.00)
Don’t Know	1.03	0.76
	(0.95)	(0.35)
Other	0.80	1.29
	(0.59)	(0.36)
Constant	0.07***	2.19
	(0.00)	(0.20)
n	822	822

*Notes*: Odds ratios and p-values shown; regressions run with survey weights and robust SEs. *p < 0.1, **p < 0.05, ***p < 0.01.

## 5. Conclusion and ramifications

In this paper I have reported on the findings of three survey experiments run to test the impacts of different types of information pertaining to donor generosity on people’s views about government aid. In the first experiment, the provision of accurate information on how little aid Australia gives had no discernible impact on Australians’ views about Australian aid giving. In the second experiment, the state of current giving was contextualised by showing participants trends in Australian aid generosity over time. Adding context in this manner produced one statistically significant finding, reducing the proportion of respondents who thought Australia gave too much aid. However, only one response category was affected and the substantive impact of the treatment was quite small. Had my analysis stopped at that point, it would have suggested that, while Australians are often poorly informed about how little aid their government gives, their attitudes cannot be readily shifted through additional information about this issue. However, the third experiment showed otherwise: contrasting Australian aid cuts to rising aid budgets in the United Kingdom moved opinions in a way that was both statistically significant and meaningful in a substantive sense. Australians’ views about aid are not intractable; however, it appears the psychological processes involved in their shifting do not involve simple calculation and reasoning.^10^ Rather, the results from these survey experiments fit with a model in which views are most easily shifted by other psychological processes. Experiment 3 contained no information that had not been included in one or other of the previous experiments except for the comparison to the United Kingdom. (Experiment 3 was explicit in mentioning aid cuts unlike Experiment 1; however, Experiment 2 also showed Australian aid cuts.) Because the treatment used in Experiment 3 contained no additional information other than a comparison to another country, the most likely causal pathway involved in its effect appears to be a desire for Australia to conform to international norms or a similar concern with international standing or the desire to be seen to be a good international citizen.

These findings fit well with studies of other aspects of human behaviour. A range of work studying domestic political behaviour has shown people to be motivated reasoners – decision makers who tend to be unpersuaded by evidence unless it fits with their existing priors (Lodge & Taber, 2013; Redlawsk, 2002; Taber & Lodge, 2006). Recent experimental work has also found motivated reasoning present in shaping people’s views about international issues (Herrmann, 2017). For these reasons, motivated reasoning is a likely explanation for the underwhelming effects of the first two treatments. By the same token, the fact that people tend to be norm conformers, or similarly concerned with the perceptions of others in matters of generosity, is well established (Ariely et al., 2009; Tankard & Paluck, 2016). Such effects appear a likely explanation for the efficacy of the treatment used in Experiment 3.

Recent work in Behaviour International Relations has begun to provide a rich empirical backdrop for theories of international politics in which key actors, including the public, are guided by complex psychological processes (Hafner-Burton, Haggard, Lake, & Victor, 2017; Herrmann, 2017). The findings of the three experiments described in this paper fit with this literature, and add to it, by demonstrating that similar complexity is present in the psychological processes influencing the ways people think about government aid.

From here there is much scope for additional work. One obvious area is to test whether the different treatments I have looked at have differing impacts on different population sub-groups. Possibly, for example, younger people, or people on the political left, or better educated people, may be affected more by the treatments I have used. The differences between my findings in Experiment 2 and those of Gilens (2001) point to the potential of treatment effects being conditional on political knowledge or interest. This should be further tested in future work.

Similarly, the most likely difference between my findings and those of Bianca Di Julio et al. (2015) is the fact that they asked survey participants for their views on aid volume after actively having previously directly corrected participants’ mistaken overestimates. As a result, their finding probably shows the effect of being shown to have been wrong by another individual, and the subsequent desire not to be seen to be unmoved by having been corrected, rather than the simple effect of new information. While this explanation seems very plausible, formally testing it would be useful.

There is also scope for learning which types of countries work well as comparators. Would, for example, Australians be as moved by comparisons to other generous aid donors such as Sweden, with which Australia has no historical ties? Learning about different comparator country effects could add to our understanding of the psychological processes that drove the results in Experiment 3. If perceived normative international communities are crucial, then it is unlikely that countries such as Sweden will be as efficacious as the United Kingdom was in this study. If, on the other hand, the motivating factor is simply the desire to be witnessed to be doing good, similar to that found in some NGO donation experiments, it may be the case that a similar effect can be obtained even with a comparator such as Sweden.

There is also scope for further research to test the efficacy of appeals to more abstract entities such as international targets and agreements. Would, for example, appeals to the promise of increased aid spending included in the Sustainable Development Goals have any impact on people’s views? Investigation of other information treatments to do with the current state of global development would also be useful. An obvious additional treatment would be information about the severity of poverty in aid-recipient countries. Work on donations to NGOs has shown need to have a clear effect on which countries people prefer their donations to go to (Hansen et al., 2014) and in instances on generosity in donations (Brañas-Garza, 2006). It will be very useful to learn if this effect can also be found with respect to aid given by governments.

For the time being, however, this paper has demonstrated that public views about aid volumes can be shifted by additional information on donor generosity. Yet, it has also shown that the information required is not the information one might expect. The psychological processes underpinning opinion change are not as simple as people readily updating their views when provided with additional facts that correct mistaken beliefs.

## Notes

1. In designing this question, different versions of the treatment were considered including using percentages and giving absolute dollar amounts. Ultimately, the wording used was chosen because it appeared to be the most readily understood. Data were for the 2015–2016 financial year. All the data on Australian aid spending used in this paper (as well as their sources and additional information) can be found at the following link: http://devpolicy.org/aidtracker/.

2. The Kaiser Family Foundation approach could be applied experimentally, using a control group that was not corrected and a treatment group that was, with subsequent differences in before and after comparisons being used to capture the effect. The impact of being proven wrong could also be mitigated by asking the follow-up question in a hypothetical way (for example, ‘if aid was x per cent of federal spending, would you say this was …’). This would allow the ability to conduct a before and after test. However, my own experimental approach involving randomisation ought to work as well, without the additional complexity, as long as randomised allocation has been correctly applied.

3. This is a slight overstatement: at least one of his treatments had a small effect.

4. Aid as a share of GNI rather than aid as a share of federal spending was used as time series data spanning many years exist for aid/GNI. Such data do not exist for aid as a share of federal spending.

5. There was a small chance that a participant might be sampled more than once; however, because the survey company’s survey population was very large compared to the samples it drew for its weekly surveys, this risk was slender and at the absolute most would have affected only one or two participants.

6. This approach was chosen for simplicity’s sake. Running multinomial logistic regressions did not change the results in any substantive manner.

7. The relationship between the control variables and views about aid found in this paper are broadly consistent with findings from other work on public opinion about aid in Australia. In all three regressions in this paper, academic education and centre-left political views were associated with more pro-aid views. This is consistent with other studies, which have found the same relationships using a range of techniques and questions. The regressions in this paper provide mixed results for gender (men are usually, but not always, less favourably disposed towards aid), age (older people are usually, but not always, less favourably disposed towards aid), and residence (urban dwellers are usually, but not always, more favourably disposed towards aid). These relationships, and similar inconsistencies, can also be found in other Australian work (for relevant work see: Wood, 2015; Wood, Humphrey Cifuentes & Pryke, 2016).

8. With parties controlled for (with no other controls included) the p-value falls to 0.08.

9. The average marginal effect is plotted. The average marginal effect is the mean of the predicted outcomes for all observations in the dataset. Calculating the marginal effects with all other variables set at their means as an alternate approach produced very similar outcomes.

10. It is possible to concoct an argument through which the information on the United Kingdom’s aid budget had an impact in a rational manner because calculating Australians feared the loss of geostrategic or economic benefits as their country lagged a ‘competitor’. This explanation, however, is unconvincing. Other survey data show that about three quarters of Australians want their country’s aid given to benefit people in poorer countries rather than to benefit their own country (Wood, 2015, p. 24). This makes competitive comparisons involving national interest an unlikely explanation for shifts in opinion.
