# Supplementary material for: Can Information Change Public Support for Aid?
Source: J Dev Stud. 2018 Jul 16;55(10):2162–76. doi: 10.1080/00220388.2018.1493194 (PMC6817318; doi:10.1080/00220388.2018.1493194)
Supplement: Supplemental Material [file FJDS_A_1493194_SM6752.pdf]

Supplementary online material – 'Can Information Change Public Support for Aid?'

This document contains full information on the experimental treatment and control questions used. It also details the key sociodemographic variables and their breakdown across treatment and control groups. At the end of this document I provide brief discussion of the balance of the survey and treatment groups.

Contents

Full survey questions 1

Detail on variables, and control and treatment group composition 3

Full survey questions

Experiment 1

Control question

Every year the Australian government provides aid money to poorer countries. Which of the following options best reflects your opinion about aid spending:
(a) the Australian government gives too much aid;
(b) the Australian government gives about the right amount of aid;
(c) the Australian government does not give enough aid;
(d) I don't know.

Treatment Question

Every year the Australian government provides aid money to poorer countries. Currently just under 1 dollar out of every 100 dollars of federal spending is given as aid. Which of the following options best reflects your opinion about aid spending:
(a) the Australian government gives too much aid;
(b) the Australian government gives about the right amount of aid;
(c) the Australian government does not give enough aid;
(d) I don't know.

Experiment 2

Control question

Every year the Australian government provides aid money to poorer countries. Which of the following options best reflects your opinion about aid spending:
(a) the Australian government gives too much aid;
(b) the Australian government gives about the right amount of aid;
(c) the Australian government does not give enough aid;
(d) I don't know.

Treatment question

Every year the Australian government provides aid money to poorer countries. Over time, compared to the size of Australia's economy, Australia's aid budget has become smaller.

This chart compares Australian aid to Australia's Gross National Income (a standard measure of the size of an economy). In 1971 Australia gave 45 cents of aid for every 100 dollars of Gross National Income. In 2015 it gave 25 cents. Which of the following options best reflects your opinion about aid spending:

(a) the Australian government gives too much aid;
(b) the Australian government gives about the right amount of aid;
(c) the Australian government does not give enough aid;
(d) I don't know.

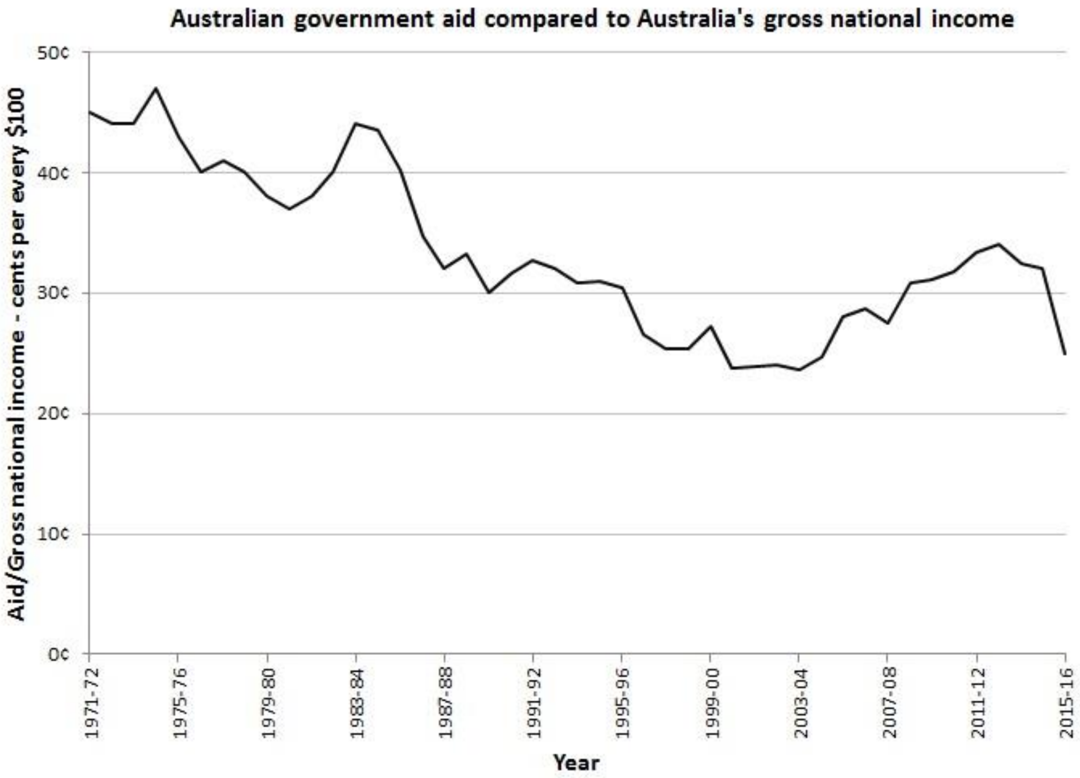

Experiment 3

Control question
Every year the Australian government provides aid money to poorer countries. Which of the following options best reflects your opinion about aid spending:
(a) the Australian government gives too much aid;
(b) the Australian government gives about the right amount of aid;
(c) the Australian government does not give enough aid;
(d) I don't know.

Treatment Question
Every year the Australian government provides aid money to poorer countries. Since 2013 Australia has reduced the amount of aid it gives. At the same time, some countries, such as the United Kingdom, have increased the aid they give. The United Kingdom now gives about 70 cents out of every $100 of its Gross National Income as aid (Gross National Income is a standard measure of the size of a country’s economy). By comparison Australia gives 25 cents out if every $100 of its Gross National Income as aid. Considering this, which of the following options best reflects your opinion about aid spending:
(a) the Australian government gives too much aid;
(b) the Australian government gives about the right amount of aid;
(c) the Australian government does not give enough aid;
(d) I don't know.

Detail on variables, and control and treatment group composition

Supplementary Table 1 shows the composition of the treatment and control groups for all three experiments. All variables are explained in the first section of the table. All variables are reported on as proportions except for income, for which the mean value is given.

Supplementary Table 1 – Sociodemographic and Political Information for Survey Participants

	Obs. Control	Obs. Treatment	Control (mean or propn)	Treatment (mean or propn)	Difference	Std. error of diff	p-value (2 tailed)
**Experiment 1**							
Male (binary; coded 1 if respondent male)	509	497	0.51	0.46	0.05	0.03	0.10
Age (binary; coded 1 if respondent over 35)	509	497	0.68	0.74	-0.05	0.03	0.07
Urban (binary; coded 1 if residing in urban area)	509	497	0.63	0.71	-0.07	0.03	0.01
Respondent income (thousands; as natural log)	427	419	4.10	4.09	0.01	0.05	0.80
Academic education (binary; coded 1 if has non-vocational tertiary education)	505	493	0.35	0.40	-0.05	0.03	0.14
Party							
Coalition (centre-right)	488	475	0.38	0.36	0.02	0.03	0.58
Labor (centre-left)	488	475	0.30	0.33	-0.03	0.03	0.29
Greens (centre-left)	488	475	0.09	0.08	0.01	0.02	0.43
Other	488	475	0.08	0.12	-0.04	0.02	0.04
Don't know	488	475	0.14	0.10	0.04	0.02	0.06
**Experiment 2**							
Male	513	499	0.50	0.47	0.03	0.03	0.37
Age (proportion > 35)	513	499	0.70	0.71	0.00	0.03	0.95
Urban	513	499	0.66	0.66	0.01	0.03	0.80
Mean income (thou; ln)	427	439	4.07	4.01	0.06	0.05	0.21
Academic education	508	491	0.39	0.38	0.01	0.03	0.83
Party							
Coalition	501	483	0.37	0.37	0.00	0.03	0.97
Labor	501	483	0.31	0.30	0.01	0.03	0.75
Greens	501	483	0.10	0.11	0.00	0.02	0.85
Other	501	483	0.11	0.10	0.01	0.02	0.67
Don't know	501	483	0.12	0.13	-0.01	0.02	0.55
**Experiment 3**							
Male	532	468	0.49	0.46	0.04	0.03	0.24
Age (proportion > 35)	532	468	0.74	0.75	-0.02	0.03	0.53
Urban	532	468	0.66	0.66	0.00	0.03	0.91
Mean income (thou; ln)	458	392	4.07	4.04	0.04	0.05	0.44
Academic education	527	464	0.38	0.38	0.00	0.03	0.99
Party							
Coalition	522	448	0.39	0.37	0.02	0.03	0.44
Labor	522	448	0.26	0.29	-0.02	0.03	0.41
Greens	522	448	0.09	0.10	0.00	0.02	0.91
Other	522	448	0.15	0.14	0.01	0.02	0.75
Don't know	522	448	0.10	0.11	-0.01	0.02	0.78

Treatment and control groups are well balanced in experiments two and three. However, in experiment one the differences in composition are statistically significant ($p<0.05$) for urban residence and the proportion of people who say they prefer an 'other' political party. In the logistic regressions reported on in the paper 'other political

party' and urban residence were sometimes statistically significant predictors of views about aid. Note, however, that controlling for the variables that were unbalanced in experiment one did not change the finding that the treatment in that experiment had no effect.
